# Process evaluation findings from Strong Hearts, Healthy Communities 2.0: a cardiovascular disease prevention intervention for rural women

**DOI:** 10.1186/s12966-024-01670-y

**Published:** 2024-10-22

**Authors:** Jacob Szeszulski, Laura J. Rolke, Priscilla Ayine, Regan Bailey, Margaret Demment, Galen D. Eldridge, Sara C. Folta, Meredith L. Graham, Alexandra L. MacMillan Uribe, Andrew McNeely, Miriam E. Nelson, Kristin Pullyblank, Chad Rethorst, David Strogatz, Rebecca A. Seguin-Fowler

**Affiliations:** 1 Institute for Advancing Health Through Agriculture (IHA), Texas A&M AgriLife Research, 17360 Coit Rd, Dallas, TX 75252 USA; 2 Friedman School of Nutrition Science and Policy, Tufts University, 150 Harrison Avenue, Boston, MA 02111 USA; 3 Bassett Research Institute, One Atwell Road, Cooperstown, NY 13326 USA; 4 Institute for Advancing Health through Agriculture (IHA), Texas A&M AgriLife Research, Department of Nutrition, College of Agriculture and Life Sciences, 498 Olsen Drive, College Station, TX 77845 USA

**Keywords:** Physical activity, Nutrition, Cardiovascular disease, Rural, Women, Ecology

## Abstract

**Background:**

Strong Hearts, Healthy Communities 2.0 (SHHC-2.0) was a 24-week cardiovascular disease prevention program that was effective in improving physical activity and nutrition behaviors and clinical outcomes among women in 11 rural New York, USA towns. This study evaluated the delivery of SHHC-2.0 to prepare the intervention for further dissemination.

**Methods:**

This process evaluation was guided by the Medical Research Council recommendations and engaged program leaders and participants (i.e., women over age 40) using quantitative and qualitative methods. The quantitative evaluation included examination of enrollment and retention data, a participant survey, and a fidelity checklist completed after classes. Descriptive and comparative statistics were used to assess implementation measures: program reach, participant attendance, dose delivered, program length, perceived effectiveness, fidelity, and participant satisfaction. The qualitative evaluation included focus groups (*n* = 13) and interviews (*n* = 4) using semi-structured guides; audio was recorded and transcripts were deductively coded and analyzed using directed content analysis and iterative categorization approaches. Comparisons across towns and between intervention and waitlist control groups were explored.

**Results:**

Average reach within towns was 7.5% of the eligible population (range 0.7-15.7%). Average attendance was 59.8% of sessions (range 42.0-77.4%). Average dose delivered by leaders was 86.4% of curriculum components (range 73.5-95.2%). Average session length was 51.8 ± 4.8 min across 48 sessions. Leaders’ perceived effectiveness rating averaged 4.1 ± 0.3 out of 5. Fidelity to curricular components was 81.8% (range 67.4-93.2%). Participants reported being “more than satisfied” with the overall program (88.8%) and the health benefits they obtained (72.9%). Qualitative analysis revealed that participants: (1) gained new knowledge and enjoyable experiences; (2) perceived improvements in their physical activity, nutrition, and/or health; (3) continued to face some barriers to physical activity and healthy eating, with those relating to social support being reduced; and (4) rated leaders and the group structure highly, with mixed opinions on the research elements.

**Conclusions:**

SHHC-2.0 had broad reach, was largely delivered as intended, and participants expressed high levels of satisfaction with the program and its health benefits. Our findings expand on best practices for implementing cardiovascular disease prevention programs in rural communities.

**Clinical trials Registration:**

www.clinicaltrials.gov #NCT03059472.

**Supplementary Information:**

The online version contains supplementary material available at 10.1186/s12966-024-01670-y.

## Background

Cardiovascular disease (CVD) is one of the leading causes of death in the United States, costing over $620 billion in 2020 alone [1–3]. CVD is more prevalent in rural communities than urban; in part, this is due to less access to prevention resources, screening/early detection, and treatment options [4–6]. Similarly, rural communities often have fewer primary care providers, less infrastructure for physical activity (e.g., community recreation centers), and limited access to nutritious food compared to urban communities [7–9]. Consequently, evidence-based CVD prevention programs are needed to improve health behaviors, improve quality of life, and reduce CVD risk factors [10, 11].

Limited CVD risk-reduction programs exist for rural populations, and implementation of these programs remains challenging [12–14]. A review of CVD prevention programs in rural communities found that contextual factors such as lack of awareness and understanding about behavioral programs, limited support from healthcare providers and social circles, and unfavorable attitudes were all barriers to engaging in CVD prevention programs [15]. Further, rural communities in the United States are heterogeneous, which suggests that some barriers and facilitators may be region specific (e.g., winter weather) or may be related to a program’s fit for that specific community [16, 17]. For example, one rural community may have a grocery store with easy access to fresh produce, whereas residents of another community may have to drive more than 30 miles to the nearest grocery store.

Strong Hearts, Healthy Communities was a CVD prevention multilevel intervention that was adapted from three evidence-based curricula [18–20] and designed with multisector community input that includes individualized CVD prevention education, social support, and civic engagement activities to improve community health environments [21]. It was also tailored for relatively isolated communities, as it included program elements that require modest resources (e.g., hand weights, yoga mats) and could be conducted in typical community settings (e.g., churches, libraries, senior centers). The program’s objective was to improve quality of life among women in rural communities and reduce CVD-related burden and health care costs [21]. The decision to focus on women was based on the need for gender-specific programming. Prior studies have shown that there are social and environmental considerations related to group cohesion, access to appropriate local resources, and other factors that necessitate health programs for women, particularly those that include exercise [22–26].

Process evaluations – systematic examinations of program implementation – are essential to refine and optimize future iterations of a program, as well as inform the delivery of other programs in similar settings [27–29].The first iteration, Strong Hearts, Healthy Communities 1.0 (SHHC-1.0), was implemented and evaluated via a community-randomized clinical trial (cRCT) in 16 rural towns (12 in Montana, USA and 4 in New York, USA). Investigators found that participants who received the program decreased body weight, improved composite CVD risk score (i.e., American Heart Association Simple 7 score), and improved biomarkers of CVD risk (e.g., C-reactive protein) compared to control participants [21]. The process evaluation for SHHC-1.0 identified several areas for improving program delivery that were incorporated into the second iteration of Strong Hearts, Healthy Communities 2.0 (SHHC-2.0) (Table 1) [30, 31]. SHHC-2.0 was a second cRCT in 11 rural New York, USA towns and saw even greater improvements and maintenance of CVD risk factors [32–35].

**Table 1:** Adaptations between SHHC 1.0 and SHHC-2.0

SHHC-2.0 Adaptation	Reason for Adaptation from SHHC 1.0
1. Addition of a scale	Participants enjoyed using Fitbit, so another technology component was added
2. Reorganization of nutrition activities	Participants requested that nutrition education be moved earlier in the curricula
3. More aerobic exercise DVDs	Participants wanted more options for lower intensity aerobic activities
4. Replication of strength training exercises	Participants requested more consistency in strength training exercises
5. Only two sessions per week	Monthly civic engagement meetings were incorporated into session content
6. Fewer snacks/participant bring snack	Program leaders faced financial and logistical challenges to providing snacks
7. Switch from an activity log to a health journal	Program leaders did not want to enter activity log data, and some participants did not find the activity logs useful
8. Some content from the leader guide was added to the participant guide	Program leaders expressed that some of the content in the leader guide would be useful to participants
9. Daily food plan with calorie goal added	SHHC-1.0 did not improve nutrition outcomes
10. More frequent goal setting reminders	Program leaders requested more time for goal setting
11. Added homework	Used to encourage participants to engage in aerobic and strength training activities outside of class
12. New content related to social support	Used to improve interpersonal environment for cardiovascular disease prevention

Note. Table adapted from Seguin et al. 2020. SHHC- Strong Hearts, Healthy Communities

The purpose of this report was to assess the fidelity and quality of SHHC-2.0 program implementation using quantitative and qualitative methods. Findings from this process evaluation will improve SHHC for wider dissemination as well as expand the literature on best practices for implementing CVD prevention programs in rural communities.

## Methods

### Study design

SHHC-2.0 was a cluster randomized trial where 11 towns were randomized to either an immediate intervention group or waitlist control group (participants received the intervention starting at 24 weeks, after the active intervention had concluded for the first group). There were a total of 14 groups; in three of the 11 towns, two groups of participants were recruited to participate on different days during the week (e.g., Group A or Group B). Participants met twice weekly for 24 weeks (i.e., six months) at 11 community locations in medically underserved areas of rural New York between 2017 and 2018. From 0 to 24 weeks, seven groups received SHHC-2.0, as well as a Fitbit and scale to track behaviors (intervention), and seven groups did not (waitlist control). From 24 to 48 weeks, the waitlist control group (*n* = 7) received the intervention but did not receive a Fitbit or scale. Inclusion criteria were: (a) identifying as female, (b) age 40 or older, and (c) meeting criteria for obesity (BMI > 30) or overweight (BMI 25–30) and currently sedentary (on average less than one 30-minute bout of physical activity per week for the last month). Exclusion criteria were: (a) not providing informed consent or not receiving authorization to participate from a healthcare provider, (b) systolic blood pressure > 160 mmHg or diastolic blood pressure > 100 mmHg, (c) resting heart rate < 60 or > 100 beats per minute, (d) cognitive impairment [36], (e) unwilling to complete surveys or be randomized, or (f) planning to participate in another health behavior change intervention in the next six months. Study design details for the SHHC-2.0 trial are available elsewhere [30].

For this process evaluation, quantitative (i.e., enrollment and retention data, participant survey, fidelity checklist) and qualitative (i.e., focus groups, interview) data were collected and compared across groups to determine any site-specific findings for both the intervention and waitlist control exposure. The waitlist control did not receive the technology component of the intervention (i.e., Fitbit and scale) and had to wait to receive the intervention, both of which may have important implications for dissemination. We used the Medical Research Council’s guidance for process evaluations of complex interventions [27, 37] to guide the selection of outcome measures (e.g., dose, fidelity, reach), data collection methods (e.g., qualitative measures to understand context), and to review recommendations for planning, designing and conducting, analyzing, and reporting process evaluations.

### Intervention description

The SHHC-2.0 curriculum contained 48 one-hour sessions over a 6-month period that included 477 curriculum components (i.e., activities) including discussions, activities, handouts, strength exercise sessions, aerobic exercise sessions, homework, and food tastings (Supplemental Table 1). Detailed methods and frameworks for the SHHC-2.0 intervention are available elsewhere [38, 39]. Briefly, sessions focused on improving individual outcomes within an ecological framework such as aerobic physical activity (e.g., walking, cardio dance), progressive strength training, and dietary behaviors. Health education activities designed to target intrapersonal (e.g., nutrition education and food tastings), interpersonal (e.g., discussions about social support), and community levels (e.g., grocery store tour, walking tour) of the socioecological models of physical activity and nutrition were also incorporated [38, 39]. Activities from a civic engagement curriculum, called Healthy Eating and Activity in Rural Towns (HEART) Club, also known as Change Club, focused on collaboratively identifying a physical activity or nutrition problem in the community, brainstorming solutions, conducting outreach to local leaders, and attempting to implement a policy, system, or environmental change [20, 40–43]. For this evaluation, we grouped curriculum components by topic. Table 2 provides an example activity for each topic: nutrition education (*n* = 97 components), session introductions (*n* = 48), aerobic exercise (*n* = 47), strength training (*n* = 43), civic engagement (*n* = 42), active homework (*n* = 38), goal setting (*n* = 36), general health education (*n* = 34), home and community environment awareness (*n* = 23), food tastings (*n* = 20), social support (*n* = 13), physical activity education (*n* = 11), self-compassion (*n* = 8), and other topics (*n* = 17).

**Table 2:** SHHC-2.0 components and examples

SHHC-2.0 Components (i.e., activities)	Example
**General**	
Introductions (*n* = 48)	**Getting Acquainted** – Each person answers ONE of the following: 1) Why is now the time for a positive change for you in your life? 2) How might your involvement in this program for you to get healthy help others around you to get healthier?”
Goal setting (*n* = 36)	**SMART Goals –** Show participants how to develop S.M.A.R.T. goals throughout the program. • *S*pecific – goal is well defined and clear • *M*easurable – ensures you will be able to track progress • *A*ttainable – goal is “do-able” • *R*ealistic – goal can be accomplished • *T*ime-based – goal can be completed in the selected timeframe
Health education (*n* = 34)	**Individual Test Results** – Review baseline test results on several topics. • Functional Fitness • Dietary Recall Results • Mindful Eating Scores
Self-compassion (*n* = 8)	**How Would You Treat a Friend?** – Discussion of how they would treat a friend who is struggling vs. how they would treat themselves.
**Nutrition**	
Nutrition education (*n* = 97)	**Fruits and Vegetables** – A handout is provided covering the importance of fruits and vegetables. Group discusses how to clean, prepare, store, and use fruits and vegetables.
Food tastings (*n* = 20)	**Vegetable Tasting** – Participants taste a variety of raw vegetables including green peppers, yellow peppers, red peppers, carrots, broccoli, jicama, and cucumber.
**Physical Activity**	
Aerobic exercise (*n* = 47)	**Aerobic Exercise** – Participants follow a 20-minute aerobic exercise video.
Strength training (*n* = 43)	**Strength Training** – Participants complete one set of full body exercises using hand weights.
Active homework (*n* = 38)	**Aerobic Exercise Homework** – Participants are instructed to complete 20 min of aerobic exercise outside of class before then next class occurs.
Physical activity education (*n* = 11)	**Essentials of Strength Training** - A handout is provided covering strength training tips and recommendations. The group lists and discusses five barriers and facilitators to strength training. The group also discusses way to overcome barriers.
**Civic Engagement**	
Social support (*n* = 13)	**Social and Environmental Influences** – Two handouts are provided: (1) tips on how to increase social support; and (2) how family and friends affect a person’s ability to be physically active and engage in healthy eating. The group discusses how family and friends support or inhibit their ability to complete strength training. They also discuss places in the community that provide opportunities to complete strength training (e.g., gyms).
Civic engagement (*n* = 42)	**Asset Mapping** – Each person identifies 3–5 assets in their network that will support an environmental change in the community (e.g., creating an outdoor walking track). The group also discusses assets in their community that can help to create an environmental change in the community.
Home and community environmental awareness (*n* = 23)	**Walk-About** – Group goes on a one-mile, facilitated walk through town to evaluate the local food and physical activity environment.

All SHHC-2.0 sessions were led by experienced health educators (e.g., Cooperative Extension agents/health educators), hereafter ‘leaders.’ Leaders attended a full day training on curriculum, a half-day training on research methods, and weekly check-in meetings to discuss implementation challenges and successes.

### Quantitative data Collection and Analysis

Quantitative data were collected via enrollment and retention data collected by the research team, a participant survey, and a fidelity checklist completed by the leader after each session. Descriptive statistics for each group were examined and intervention (0 to 24 weeks) and the waitlist control (24 to 48 weeks) samples were pooled as part of the primary analysis but presented separately where appropriate. Analyses were conducted using STATA SE/17.0 (Stata Corp LLC, College Station, TX, USA) and SAS (SAS Institute, Cary, NC, USA). The following measures – with their definition, data collection method and analysis – were examined for the quantitative component of this process evaluation.

*Reach.* We defined reach as the proportion of age, gender, and weight eligible individuals within a town enrolled in the intervention [44, 45]. Enrollment and retention data were collected by the research team. For each town, the number of SHHC-2.0 participants (numerator) was divided by the estimated number of eligible women (denominator). The denominator was based on 2016 census data for women over age 40, multiplied by the age-adjusted rate for overweight or obese adults in New York in 2016 from the Behavioral Risk Factor Surveillance System [46, 47].

*Participant Attendance.* We defined participant attendance as the proportion of SHHC-2.0 sessions attended. Leaders collected and reported participant attendance via the fidelity checklist. Attendance was calculated as the average percent of sessions attended for each participant who attended at least one intervention session and then averaged by group.

*Dose Delivered.* Dose delivered was defined as the amount of intervention delivered by leaders (e.g., completeness) [44, 48]. Leaders reported dose delivered via the fidelity checklist. Dose delivered scores were determined by scoring each curricular component as: 1 = covered or 0 = not covered. The proportion of curricular activities that were delivered across all sessions was calculated by group and then averaged across all groups. Additionally, we examined dose delivered by content area (e.g., aerobic activity, strength training) and sample (i.e., intervention and waitlist).

*Fidelity.* Fidelity was defined as the extent to which the intervention was delivered as planned (i.e., quality) [44, 48]. Leaders recorded if they had completed each curriculum component (*n* = 477) via the fidelity checklist. Each session had a certain number of components and those components were scored as delivered as: 1 = yes, covered as prescribed, 0.5 = yes, covered with changes, or 0 = not covered. The introduction, aerobic activity, and active homework were measured as 1 = yes, covered as prescribed or 0 = not covered. Scores for each curricular component were then added and divided by the total number of recorded components for each group and averaged across all groups.

*Program Length.* Program length was defined as the total number of minutes spent on each session and on SHHC-2.0 across all sessions. Leaders reported the amount of time spent on each curricular component via the fidelity checklist. We calculated the time for each curricular component and added all components for each session and the whole curriculum. The average time to deliver SHHC-2.0 in total and for each session were calculated.

*Perceived Effectiveness.* Perceived effectiveness was defined as the subjective likelihood that the program had an impact on intended outcomes. Leaders reported perceived effectiveness via the fidelity checklist that asked them to rate how effective the session was for their participants (i.e., 1 = very ineffective to 5 = very effective). Perceived effectiveness scores were averaged across each session and across all sessions for each group.

*Program Satisfaction.* Program satisfaction was defined as the extent to which a program meets participants’ needs and expectations. Surveys were administered to SHHC-2.0 participants after 12 weeks and 24 weeks to assess participant satisfaction with the program, specific program components, individual health changes, and benefits of participation using a 5-point Likert scale (i.e., 1 = not at all satisfied to 5 = very satisfied). Program satisfaction scores were averaged across all participants, by group (intervention or waitlist control), and at each timepoint.

### Qualitative data Collection and Analysis

Following program completion, we verbally invited all participants to attend an in-person focus group at their SHHC-2.0 program site and conducted at least one focus group per site. We also conducted separate interviews with several site leaders (*n* = 4). Focus groups and interviews were scheduled for 60–90 min and participants received a $25 gift card for their participation. We developed a focus group and interview guide that inquired about characteristics of SHHC-2.0, impacts of the program (e.g., health outcomes), and external barrier and facilitators that impacted program delivery. Focus groups and interviews were led by a member of our research team (MLG) who did not deliver the program to participants and who did not code the transcripts or review and select quotes. SHHC-2.0 leaders were not present at the focus groups. Participants were audio recorded, recordings were transcribed verbatim and analyzed using deductive directed content analysis and iterative categorization approaches [49, 50]. A codebook was developed to align with the guides. Codes were designed to help identify psychosocial benefits, behavior changes, health changes, and barriers and facilitators to participation in physical activity, healthy eating, and SHHC-2.0 sessions, as well as provide an understanding of participants’ satisfaction with leaders, program structure, and evaluation processes. Two researchers (LJR, PA) concurrently coded three focus group transcripts and one interview transcript and met to discuss similarities and differences in their coding process. When discrepancies occurred between coders, the two researchers discussed the codes to reach consensus. Notes from the discussions were used to make improvements to the codebook and subsequent coding processes. One coder (LJR) coded the remaining transcripts.

Following completion of the coding process, queries were performed for quotes assigned to each deductive code. A third researcher (JS) reviewed excerpts from the queries, noted general topics, salient findings, and key points in one-quarter page to one-page written summaries. Key quotations that supported summaries were also identified. Summaries and supporting quotations were reviewed by all researchers, edited to clarify the most important points, and finalized themes were produced. Finally, one researcher (AM) reviewed themes to identify differences between intervention and waitlist control groups by checking the number and content of the quotes from each group. We used NVIVO 11.6.1 (QSR International, Doncaster, Australia) to perform coding, as well as to organize and identify codes.

## Results

A total of 182 women were enrolled in the SHHC 2.0 study across 11 towns (14 groups). Of the 182 women enrolled, 149 (82%) (intervention: *n* = 85 of 87, 98%; waitlist control: *n* = 64 of 95, 67%) completed at least one SHHC-2.0 session, 107 (59%) (58 intervention; 49 waitlist control) completed a program satisfaction survey at the end of SHHC-2.0 and 102 (56%) participated in one of 13 focus group (6 intervention focus groups; 7 waitlist control focus groups). Demographic characteristics were reported for all participants, as well as separately for the quantitative and qualitative measures (Table 3).

**Table 3:** SHHC-2.0 demographic characteristics of participants

		All	Survey Completers	Focus Group Attendees
Participants, n (%)		*n* = 182	*n* = 104 (57.1)	*n* = 102 (56.0)
	Age, y ± SD	57.2 ± 9.0	58.2 *±* 9.8	57.1 *±* 8.7
	Weight, kg ± SD	96.7 ± 21.1	94.1 *±* 19.7	96.2 *±* 21.0
	BMI, kg/m^2^ ± SD	36.7 ± 7.8	35.8 *±* 7.3	36.3 *±* 7.7
Weight status, n (%)		*n* = 182	*n* = 104	*n* = 102
	Overweight	26 (14.3)	16 (15.4)	15 (14.7)
	Obese Class 1	63 (34.6)	42 (40.4)	38 (37.3)
	Obese Class 2	47 (25.8)	24 (23.1)	25 (24.5)
	Obese Class 3	46 (25.3)	22 (21.2)	24 (23.5)
Race/ethnicity, n (%)		*n* = 168	*n* = 100	*n* = 98
	White, non-Hispanic	164 (97.6)	97 (97.0)	96 (98.0)
	Non-white or Hispanic	4 (2.4)	3 (3.0)	2 (2.0)
Household income, n (%)		*n* = 162	*n* = 97	*n* = 95
	<$25,000	29 (17.9)	17 (17.5)	12 (12.6)
	$25,000–50,000	37 (22.8)	23 (23.7)	19 (20.0)
	>$50,000	96 (59.3)	57 (58.8)	64 (67.4)
Relationship status, n (%)		*n* = 171	*n* = 102	*n* = 100
	In a relationship	116 (67.8)	68 (66.7)	70 (70.0)
	Not in a relationship	55 (32.2)	34 (33.3)	30 (30.0)
Education, n (%)		*n* = 172	*n* = 103	*n* = 101
	High school or less	26 (15.1)	16 (15.5)	15 (14.9)
	Some college/technical or vocational school	35 (20.3)	16 (15.5)	12 (11.9)
	College graduate	63 (36.6)	42 (40.8)	42 (41.6)
	Postgrad/professional	48 (27.9)	29 (28.2)	32 (31.7)
Overall health, n (%)		*n* = 175	*n* = 103	*n* = 101
	Excellent/very good	46 (26.3)	15 (14.6)	17 (16.8)
	Good	99 (56.6)	58 (56.3)	54 (53.5)
	Fair/poor	30 (17.1)	30 (29.1)	30 (29.7)

*Notes.* BMI – Body mass index; SHHC – Strong Hearts Healthy Communities; SD – Standard deviation.

### Quantitative results

*Reach.* The average reach of SHHC-2.0 was 7.5% of eligible women in each town (6.5% intervention; 11.0% waitlist control). Reach ranged from 15.7% in the smallest town to 0.7% in the largest town (Table 4). In towns where there were two SHHC-2.0 sessions, reach was higher (10.1%) than when there was only one session per town (5.9%).

**Table 4:** Process evaluation results by SHHC-2.0 Group (*n* = 14)

Site (Town)	Reach ^a^	Sessions Attended (%)	Dose Delivered ^b^ (%)	Fidelity ^c^ (%)	Session length, mean ^d^			Total Session (hrs.)	Session effectiveness, mean (Range 1–5)
**County One**									
Town 1 (1)	2.8%	57.6%	95.2%	93.2%	56.6 ± 9.7			45.3	3.9
Town 2 (2)	1.6%	63.7%	94.8%	91.6%	58.0 ± 7.3			46.4	4.0
**County Two**									
Town 3 – Group A (1)	7.3%	61.1%	87.5%	84.9%	54.1 ± 9.8			43.3	4.2
Town 3 – Group B (1)		63.1%	87.4%	85.1%	52.3 ± 12.8			41.9	4.2
Town 4 – Group A (2)	14.6%	61.6%	92.3%	89.5%	57.3 ± 7.8			45.8	4.3
Town 4 – Group B (2)		42.0%	91.1%	88.2%	56.6 ± 9.7			45.6	4.3
**County Three**									
Town 5 – Group A (1)	8.3%	66.9%	77.3%	71.2%	43.2 ± 13.8			32.3	4.0
Town 5 – Group B (1)		53.6%	73.9%	68.0%	38.5 ± 14.4			26.9	4.1
Town 6 (2)	12.9%	72.5%	74.8%	68.8%	43.0 ± 11.5			33.7	4.8
Town 7 (2)	15.7%	56.9%	73.5%	67.4%	42.1 ± 16.4			33.7	4.2
**County Four**									
Town 8 (1)	3.9%	48.3%	88.9%	85.4%	54.9 ± 9.7			43.0	3.7
Town 9 (2)	0.7%	60.0%	87.4%	84.5%	58.0 ± 7.3			43.2	3.7
**County Five**									
Town 10 (1)	7.3%	77.4%	94.7%	85.4%	51.7 ± 13.1			41.3	4.1
Town 11 (2)	2.4%	53.4%	91.0%	81.8%	54.6 ± 9.8			43.7	4.5

*Notes.* (1) denotes Group 1 (intervention + Fitbit and scale) and (2) denotes Group 2 (waitlist control). ^a^ Number of SHHC-2.0 intervention participants divided by the total number of eligible women in each town multiplied by 100. ^b^ Percentage of curriculum components delivered as prescribed or partially prescribed. ^c^ Percentage of curriculum components delivered in any form. ^d^ average length of sessions (minutes) and standard deviation

*Participant Attendance.* Of the 182 women enrolled in the study, 33 (18.1%) did not participate in a single SHHC-2.0 session, and most women who did not participate were from the waitlist control group (93.5%). For the 149 women who attended at least one session (85 intervention; 64 waitlist control), average attendance was 59.8% (60.5% intervention; 58.8% waitlist control) and ranged from 42.0 to 77.4% across groups (Table 4). Eighteen participants (12.1%) attended > 90% of sessions (12.9% intervention; 10.9% waitlist control). An additional 34 participants (22.8%) attended 75.0–89.9% of sessions (25.9% intervention; 18.8% waitlist control). Twenty-four participants (16.1%) attended < 25.0% of sessions (16.5% intervention; 15.6% waitlist control). Average attendance was 65.8% of sessions from 0 to 12 weeks (68.0% intervention; 62.9% waitlist control) and 53.7% of sessions for 12–24 weeks of the program (53.0% intervention; 54.6% waitlist control).

*Dose Delivered.* The average dose delivered across all groups was 86.4% of curricular components (86.4% intervention; 86.4% waitlist control), with a range of 73.5–95.2% across groups. Physical activity components (aerobic activity, physical activity education, strength training, and active homework) had some of the highest delivery rates (Table 5). The average dose delivered for aerobic activity was 98.9% of curricular components (intervention 99.1%; waitlist control 98.8%) and ranged from 93.6 to 100.0% by group. There were multiple options available for aerobic activities; walking outdoors (*n* = 204 times, 31.9%) and indoors (*n* = 205 times, 32.1%) were chosen most frequently by leaders. The average dose delivered for physical activity education was 96.4% (range 87.5–100.0% across groups), strength training was 96.8% (range 91.2–100.0% across groups), and active homework was 93.3% (range 80.0–100.0% across groups). Average dose delivered only differed by 5% or more for intervention vs. waitlist control on two component categories: self-compassion (95.9% intervention; 86.2% waitlist control) and social support (86.9% intervention; 97.6% waitlist control).

**Table 5:** Dose delivered and fidelity by curriculum activity category

		Mean Dose Delivered (%)			Mean Fidelity (%)		
	Total # of Components	Pooled-sample	Intervention group	Waitlist group	Pooled-sample	Intervention group	Waitlist group
All Curriculum Components	477	86.4%	86.4%	86.4%	81.8%	81.9%	81.7%%
*Component Category*							
Introduction	48	67.2%	65.8%	68.7%	-	-	-
Goal Setting	36	76.4%	75.1%	77.7%	68.0%	**65.5%**	**70.5%**
General Health Education	34	84.7%	86.0%	83.4%	74.9%	74.3%	75.5%
Self-Compassion	8	91.1%	**95.9%**	**86.2%**	87.4%	**93.2%**	**81.6%**
Nutrition Education	97	63.5%	62.7%	64.3%	58.5%	57.8%	59.2%
Recipes / Tastings	20	78.2%	79.0%	77.4%	76.9%	77.9%	75.9%
Aerobic Activity	47	98.9%	99.1%	98.8%	-	-	-
Strength Training	43	96.8%	97.3%	96.3%	87.3%	89.7%%	84.8%
Active Homework	38	93.3%	93.3%	93.3%	-	-	-
Physical Activity Education	11	96.4%	96.9%	96.0%	90.9%	91.9%	89.9%
Social Support	13	92.2%	**86.9%**	**97.6%**	88.4%	**82.1%**	**94.7%**
Civic Engagement	42	77.3%	79.5%	75.2%	74.3%	**76.9%**	**71.7%**
Environment	23	84.9%	85.0%	84.9%	82.0%	81.4%	82.6%

*Notes.* As 17 curriculum components could not be categorized, components within categories do not add up to 477. Values are bolded when a five or more-percentage difference exists between intervention and waitlist control groups

*Fidelity.* Across all groups, fidelity to the curriculum was 81.8% (81.9% intervention; 81.7% waitlist control), with a range from 67.4 to 93.2% for each SHHC-2.0 group. Similar to dose delivered, the physical activity components were delivered with the highest fidelity (Table 5). Strength training exercises were described as: intro exercises (*n* = 4), upper body exercises (*n* = 14), full body exercises (*n* = 13), core exercises (*n* = 6), and lower body exercises (*n* = 6). Average fidelity to the strength training components was 87.3%, (89.7% intervention; 84.8% waitlist control) with a range of 66.3–95.3%. The group with the lowest fidelity completed strength training regularly, but for about half the sessions, the group chose additional exercises provided within a supplemental list from the research team.

*Program Length.* Across all sessions, there were 20 (3.0%) without time information for the session or time estimates of less than 20 min, which we excluded from the analysis. The average group met for approximately 40.4 h over the 48 sessions in the 24-week period (39.1 h for the intervention; 41.7 h for the waitlist control). The average session was approximately 51.8 min ± 4.8 min in length (50.0 ± 11.9 min for the intervention; 53.0 ± 9.9 min for the waitlist control).

The component categories with the lowest dose delivery were nutrition education and the introduction content. The average dose delivered for nutrition education was 63.5% (62.7% intervention; 64.3% waitlist control; range from 59.8 to 65.7% across groups). The introduction, a short paragraph read aloud to explain the session’s activities, had the largest range of delivery between SHHC-2.0 groups (range from 4.3 to 100.0% by group). The average dose delivered was 67.2% (intervention 65.8%; waitlist control 68.7%).

*Perceived Effectiveness.* Leaders scored SHHC-2.0’s average effectiveness as 4.1 ± 0.3 out of 5 across all sessions (4.0 ± 0.2 intervention; 4.3 ± 0.4 waitlist control) and across groups (range 3.7 to 4.8). Although all sessions were rated highly, the two most highly rated sessions focused on Mindful Eating and Activity (e.g., keeping a positive mindset) and Social Environment (e.g., communicating with others about weight control).

*Program Satisfaction.* At the end of the program, 88.8% of participants were “more than somewhat satisfied” with the program (88.9% intervention; 87.7% waitlist control) and 72.9% were “more than somewhat satisfied” with the health benefits they obtained from the program (70.7% intervention; 75.5% waitlist control). More than 80.0% of participants were “more than somewhat satisfied” with the lessons (89.6% intervention; 87.2% waitlist control), discussions (91.4% intervention; 93.6% waitlist control), healthy snacks (82.7% intervention; 89.6% waitlist control), strength training (86.2% intervention; 89.6% waitlist control), and aerobic exercises (84.5% intervention; 81.6% waitlist control). Participants reported being “more than somewhat satisfied” with SHHC-2.0’s benefits, including more physically active (82.7% intervention; 83.7% waitlist control), physically stronger (82.7% intervention; 83.6% waitlist control), more energy (75.9% intervention; 77.6% waitlist control), eating healthier (63.8% intervention; 69.4% waitlist control), and better sleep (50.9% intervention; 61.2% waitlist control). Satisfaction at the end of the study (24 weeks) was not substantially different than at the 12-week assessment.

### Qualitative results

Thirteen focus groups were held with a median size of 7 participants (range 2 to 20), and four leader interview were conducted. Qualitative analysis revealed that participants: (1) Gained new knowledge and enjoyable experiences; (2) Perceived improvements in physical activity, nutrition, and/or health; (3) Continued to face some barriers to physical activity and healthy eating, however those relating to social support were reduced; and (4) Rated leaders and the group structure highly, with mixed opinions on the research elements. No differences were found between intervention and delayed intervention groups; thus, results were presented together.

*Theme 1: Participants gained new knowledge and enjoyable experiences.* For physical activity, participants discussed gaining new knowledge about types of exercises (e.g., strength training exercises) and the safety of those exercises (e.g., rating of perceived exertion, how to choose the right weights) (Table 6 - Quote 1). Specifically, they enjoyed completing physical activity as a group, seeing improvements in their quality of life, and making small changes to their daily routines (Quote 2). For nutrition, participants discussed gaining new knowledge about foods that they had never tried before (e.g., lentils, quinoa, avocado, kale, cauliflower rice, rainbow carrots) as well as some of the benefits of those foods (e.g., fiber makes you feel full, glycemic index) (Quotes 3 and 4). In particular, they enjoyed food tastings and when they went grocery shopping for recipes (Quotes 5 & 6). For the civic engagement project, participants discussed gaining new knowledge of resources that existed in their community (e.g., walking opportunities). However, participants did not enjoy the civic engagement components as much as other components, as they reported already being extremely involved in the community, currently, or in the past, and most people saw SHHC-2.0 as something that they were doing for themselves (Quote 7).

**Table 6:** Qualitative themes and supporting quotes

**Theme 1**: Participants gained new knowledge and enjoyable experiences.			
Quote 1	I think learning the core exercises and proper use of weights and stuff will be a long-term benefit for me. • *Town 8 Participant*		
Quote 2	… but if you stroll around every couple of hours for 3 min [it] is not a bad thing so. you know. that was something that I was aware of, that I had the freedom to…add physical activity, so the class kind of reminded me of that, which was great. • *Town 5 Participant*		
Quote 3	I think one of the things that was probably the most fun for me is that…the things that people talked about, the different foods that they were eating that they’d NEVER tried and- KALE, I love kale. Most people have never had kale. • *Town 6 Participant*		
Quote 4	I do notice that certain things I eat now, it does make me stay fuller longer and it’s healthier for me. Things I didn’t know, I mean, I never would have tried black bean anything… • *Town 3 Participant*		
Quote 5	I think that getting them involved to do it [make the recipes] was very important because a lot of them have never had or made…those food choices … you know, everybody loves food. *Towns 5*,* 6*,* and 7 Leader*		
Quote 6	I definitely would have done two snacks, because I think that made me pick new things at the grocery store. Quinoa. I didn’t even know what that was until we brought it in. So, I think if we did it more often maybe. • *Town 11 Participant*		
Quote 7	It’s funny, because this turned out to be a community engagement project, but I think of it more as for myself. • *Town 11 Participant*		
**Theme 2**: Participants perceived improvements in physical activity, nutrition, and/or health			
Quote 8	Interviewee 1:You could even help with your porch. Interviewee 2:Yes. We actually ripped down a porch and are rebuilding. I kind of was like, “Ha-ha, look at me,” and my husband was like, “Yeah. Okay.” • *Town 9 Participants*		
Quote 9	I was very satisfied. I knocked off a few pounds and then…my clothes felt more comfortable. • *Town 8 Participant*		
Quote 10	We did not have a great deal of success with weight loss but we had a phenomenal success…I think all of us can say this…with mobility, with balance, muscle. • *Town 6 Participant*		
Quote 11	We had one, like, shining-star success story who said that the program saved her life. • *Towns 8 & 9 Leader*		
**Theme 3**: Participants continued to face some barriers to physical activity and healthy eating, however those relating to social support were reduced.			
Quote 12		As much as we wanted a lifestyle change or [to] maybe lose weight or something, I think we were more looking for the camaraderie…the commonality of…aging early. • *Town 3 Participant*	
Quote 13		If [participant name] stops going, I won’t go there by myself. • *Town 7 Participant*	
Quote 14		My [Town 10] group and my [Tonw 11] group both really enjoyed the opportunity to get together and exercise with supportive women • *Town 10 and 11 Leader*	
Quote 15		If somebody said, “Can you take even an hour and fifteen minutes [for the session],” I might have re-thunk it, you know, because I would have been like, “Ooh, an hour, and fifteen minutes?” But… after a month or two, I would have been like, “Hell, yeah. I’d stay for an hour and fifteen minutes.” • *Town 11 Participant*	
Quote 16		“Oh, we have an older persons’ yoga class” and I’m thinking, that’s not what I need … but I need cardio where I’m moving, I can’t get down on the ground and put my right leg around my head and those things, but I can do [SHHC-2.0]. • *Town 3 Participant*	
Quote 17		“Everybody is going to be there. I should go,” you know? Because, like, I enjoyed the camaraderie of the group and just felt a little accountable to go see everyone and do our routine. • *Town 11 Participant*	
Quote 18		I don’t feel disconnected, but I feel like we never [have]… even gone around the table and said where we are coming from. That was my suggestion for tonight. Go around the table and say your name, where you are from, and what you do for a living or what your interests are. We never really did that. • *Town 1 Participant*	
Quote 19		We talked about exercise and diet and [things] like that, and I learned a lot from everybody, and that was really helpful. It helps to have a good group that you can sit there and talk like this and feel comfortable. • *Town 2 Participant*	
**Theme 4**: Participants rated leaders and the group structure highly, with mixed opinions on the research elements.			
Quote 20		And she always sent back very positive messages, like if I couldn’t make a meeting, it would always be, “Well, stay strong!” You know…and “Hope you’re doing well, stay strong,” …whatever you would do…”Remember to exercise,” “Remember to eat well.” • *Town 4 Participant*	
Quote 21		The best thing about this course were your instructors and they were not given enough time to share their wealth of information. • *Town 1 Participant*	
Quote 22		I’m just gonna say this really, really quickly, but those dietary recalls, if you want them accurate, they need to be less cumbersome. The fact that it takes me five minutes to tell you about the supplements that I have every day, the same weight, the same amount, the same thing, the fact that every time I have water, I have to tell you it’s tap water in five steps. • *Town 5 Participant*	
Quote 23		I think the questionnaire, because to me…the questionnaire that took hours to complete, it really didn’t have anything to do [with SHHC-2.0]. • *Town 6 Participant*	
Quote 24		The program can be kind of overwhelming, just all of the data collection that’s constantly happening. • *Town 8 & 9 Leader*	
Quote 25		The problems for me were that there were times for me that my computer was down at home or something, and I couldn’t get to the website to do it when I wanted to, and I was thinking…for a lot of people in rural environments like was already mentioned, you know, internet doesn’t always work the best. • *Town 5 Participant*	

*Theme 2: Participants perceived improvements in physical activity*,* nutrition*,* and/or health.* For physical activity, SHHC-2.0 helped participants to start a new exercise routine, get out and walk more frequently, or try new activities (e.g., joining a fitness center, running a 5 K). They also described doing more types of physical activity in their day-to-day lives (e.g., carrying their grandchildren, building a deck, raking leaves) or just adding small bits of activity throughout their day (e.g., taking walking breaks at work) (Quote 8). For nutrition, SHHC-2.0 helped participants switch the types of foods they were eating. For example, participants described making new recipes that they learned during the program (e.g., black bean burgers), drinking more water instead of sugar-sweetened beverages or coffee, and making healthy substitutions in recipes (e.g., whole wheat instead of white bread). A few participants also discussed limiting certain foods (e.g., chips, red meat, bread); however, this was less common. For health benefits, most participants discussed weight loss, regardless of if they lost weight or not. Many participants did lose weight, from a few pounds or a clothing size up to 30 pounds (Quote 9). However, some participants maintained their weight and one person even mentioned gaining ten pounds. Outside of the discussions around weight loss, participants described feeling stronger, having better cardiorespiratory fitness, improving balance, being able to do more activities of daily living (e.g., raking leaves, walking upstairs) and reductions in CVD risk factor parameters (e.g., blood pressure, cholesterol) (Quote 10 & 11).

*Theme 3: Participants continued to face some barriers to physical activity and healthy eating*,* however those relating to social support were reduced.* Generally, participants described the cold weather and social isolation that occurs during the winter months, work-related barriers (e.g., limited time, no breaks, social norms), community barriers (e.g., no sidewalks, only fast food restaurants, cost of gym memberships and healthy foods), and in some cases, lack of social support from immediate family members, as barriers to participation in physical activity and healthy eating. However, regarding social support, a few participants stated that their families encouraged them to attend SHHC-2.0 and that coworkers and friends were supportive of their lifestyle changes. Pre-existing physical limitations – knee and back injuries, heart problems, injury, fibromyalgia, breast cancer – also came up frequently as a barrier that prevented or limited participants’ engagement in physical activity.

The SHHC-2.0 program removed some social support barriers because other SHHC-2.0 participants provided a support system (Quote 12), and SHHC-2.0 provided accountability for participants to engage in exercise, which they enjoyed (Quote 13 & 14). Some participants expressed challenges to attending SHHC-2.0, as well as to sustaining group sessions beyond the study. Key barriers to attending SHHC-2.0 included long work hours, multiple other responsibilities (e.g., second job, child’s school), lack of transportation, and the long distances that participants needed to travel to get to the site where SHHC-2.0 was being implemented (e.g., over 1 h). Limited time was also mentioned as a barrier; however, several participants expressed that once they started coming, they wanted more time for discussion, goal setting, and activities, as they did not feel like one hour was long enough (Quote 15). Most participants wanted to continue meeting as a group once the study was over. However, finding someone to continue to organize meetings, financial and insurance requirements to rent meeting spaces, and finding a central location were all challenges to sustaining sessions beyond the study. Switching to an existing class (e.g., senior yoga) was mentioned by several participants, but many felt that existing classes were not tailored to meet their needs or that they would not feel comfortable in those classes (Quote 16).

Overall, participants appreciated the social support that the group format provided. Group discussions helped anchor educational concepts, as well as provide support, accountability, and friendship (Quote 17). Different groups had varying levels of connection; several participants stated that they wished there was more time at the beginning of SHHC-2.0 to get to know one another, and that even now, in the post-intervention focus groups, they did not know the names of everyone in their group (Quote 18). Still, participants felt like they had bonded with one another. They also stated that 6 months and about 10 people were the right numbers to develop group cohesion. Participants acknowledged that it was nice to get to know new people within their community who had the same issues as them and who were their age and fitness level (Quote 19).

*Theme 4: Participants rated leaders and the group structure highly*,* with mixed opinions on the research elements.* Participants described program leaders as outstanding and critical to SHHC-2.0 success. Attributes that participants enjoyed in the program leaders included being upbeat, positive, generous with their time, able to individualize material, organized, empathetic, and knowledgeable (Quote 20). In particular, many participants mentioned how well the leaders described the strength exercises and provided modifications. One participant even described the leaders as the best part of the program, and several participants felt strongly that leaders were not given enough time to share their knowledge (Quote 21).

Participants also reported liking some aspects of the research elements, especially when they saw improvements in their health outcomes: blood pressure, cholesterol, and weight. In particular, the participants were divided about using weight as an outcome – some people liked to focus on other health behaviors, and some wanted weight measured and discussed weekly. Participants felt like positive changes in health outcomes validated their efforts in SHHC-2.0; however, as one participant stated, seeing no changes or worsening health outcomes could be “devastating” and several participants were worried about how physical ailments (e.g., knee injury, foot surgery) affected their results. Dietary recalls and the baseline survey were reported as strongly disliked by participants due to the time they took to complete (Quotes 22, 23, and 24). Further, challenges with internet access in rural areas made the online assessments hard to complete or costly (Quote 25). Some participants thought that three assessment timepoints (i.e., baseline, midpoint, end) were the right amount, whereas others wanted more frequent checks on their weight so they could modify their plan if they were not seeing improvements. Participants also wanted more explanation of their personal data.

## Discussion

Prior research demonstrates that SHHC-2.0 has a positive impact on the physical activity, diet, weight, and other CVD risk factors among women in rural regions [32–35]. Rural health disparities are exacerbated by direct and indirect factors that affect behaviors, such as geographic distances and transportation barriers and limited access to healthy foods, physical activity, and healthcare [51]. A survey of nearly 1500 rural stakeholders nationwide indicated that healthcare access and quality, overweight and obesity, nutrition and healthy eating, and preventive care were all in the top 10 priorities for rural America [52]. This process evaluation shows that the program was perceived positively by rural participants and most elements were implemented well; yet, implementation challenges remain regarding attendance and balancing fidelity to the program with adaptations to support barriers to implementation.

SHHC-2.0 reached an average of 7.5% of eligible women. Notably, in towns where there were two SHHC-2.0 sessions, the reach was about double that of towns with one session (10.1% vs. 5.9%). As a second session was only offered based on enrollment, this data suggests that there is a demand for SHHC-2.0 within rural communities. Still, we may have underestimated reach (see limitations). Further, current demographic trends suggest that rural communities continue to shrink in size, and as a result, many rural health services (e.g., doctors, mental health providers) are also subsequently diminishing [16, 17]. Likewise, SHHC-2.0 could not be conducted in some of the smallest towns with fewer amenities, and some participants still had to drive a substantial distance to attend classes. Thus, creating tailored CVD prevention programs outside of traditional healthcare systems that are well-received by the community is an immediate need of critical importance, and identifying resources in rural communities that can support these classes is vital. At the end of SHHC-2.0, most participants were “more than somewhat satisfied” with the program (> 85%) and “more than somewhat satisfied” with the benefits they received from the program (> 70%). Furthermore, qualitative results suggested that existing programs within the community do not meet the needs of participants. With continued tailoring and the development of strategies to improve implementation processes (i.e., implementation strategies) [53, 54], SHHC has the potential to be widely disseminated for CVD prevention in rural towns across the United States.

Average attendance was about 60% (65.8% from 0 to 12 weeks and 53.7% from 12 to 24 weeks), and 69% of participants attended > 50% of classes, which aligns with or is slightly lower than other rural community-based health promotion programs but are still promising given SHHC-2.0’s length (24 sessions) [33, 55]. Qualitative findings suggested that barriers to attending SHHC-2.0 exist and are similar to barriers found in other rural communities (e.g., limited time, long work hours, multiple other responsibilities) [56] One way to balance attendance challenges with participants’ desire for more curricular content may be the use of a flexible, online, or hybrid implementation model [57, 58]. However, social support was a major facilitators of attendance, and some participants had no or limited access to the internet. Future iterations of SHHC-2.0 or other rural health programs should explore using multiple modes of delivery, as well as developing asynchronous online curricular components that continue to facilitate social support.

Lack of fidelity to specific components resulted in participants missing some beneficial information (e.g., 64% of dose delivered for nutrition education). Lower fidelity for some curricular components may have occurred because the SHHC-2.0 implementation guide explicitly tells leaders to prioritize in-class physical activity if they have limited time. Fidelity and dose delivered are important aspects of program implementation that have the potential to negatively impact the effectiveness of SHHC-2.0 [27, 37]. However, program drift (i.e., differential implementation/fidelity) can also have a positive impact on SHHC-2.0 if changes better align with participants’ needs, knowledge, attitudes, or health behaviors [59]. Qualitatively, participants believed that leaders needed more time, including time to convey their knowledge and to cover all the materials in the curriculum. However, the average session length was about 50 min, 10 min less than the recommended class length (i.e., 60 min). Often these 10 min were spent problem solving with individual participants about scales, homework, discussion, etc. Thus, it could have had a positive impact on outcomes, despite not being a part of the curriculum.

Future interventions should consider the balance of content and time to enhance participant experience and reduce implementation burden. A better understanding and specification of how each curricular component affected outcomes, via a logic model, could also help improve the overall efficiency of SHHC-2.0. Furthermore, adaptive designs or factorial trials that evaluate individual intervention’s components are needed to identify components that are time-varying, as well as identify components that could be modified or removed [60–62].

### Limitations

Our sample was primarily white, which is comparable to the population at large in this region of the United States. However, many rural populations are more racially and ethnically diverse; [47] thus, SHHC-2.0’s results may not be generalizable to other populations in other rural areas. Since we could not determine the total eligible population based on all inclusion and exclusion criteria (e.g., permission from primary care provider, cognitive impairment), reach may be underestimated; we did our best to evaluate reach based on all eligibility criteria that could be assessed in the wider population (e.g., age, obesity status). There were also a few SHHC-2.0 sessions (3.0%) without any time information for the session or time estimates less than 20 min. It is unclear why these data were missing or incomplete; however, such a small amount of missing data is unlikely to substantially impact findings. Additionally, fidelity was rated as follows: 1 = yes covered as prescribed, 0.5 = covered with changes, and 0 = not covered. However, not all adaptations may affect outcomes equally. Future work should consider reporting adaptations using a structured framework that addresses the type and potential impact of the adaptation on the intended outcomes (e.g., FRAME – Framework for Reporting Adaptations and Modifications to Evidence-based interventions) [63]. Researchers conducted the focus groups, which may have led to some social desirability bias and overly positive qualitative findings. However, leaders involved with implementation were not present for the focus groups, multiple researchers were involved in the coding process, and both leaders and participants reported similar experiences, which helps to triangulate qualitative findings. Finally, this study included time-consuming research data collection; this may have impacted program delivery (e.g., attendance, satisfaction). However, many participants also appreciated seeing changes in their health values as part of the SHHC-2.0 program. Researchers should carefully consider the balance between study data and participant burden, especially focusing on measurements that can add value to participants’ experiences with the program.

### Strengths

Use of both quantitative and qualitative process measures allowed for a more detailed description of factors affecting implementation and indicated potential opportunities for improving the delivery of SHHC-2.0 or other similar CVD prevention programs. A large proportion of participants provided qualitative feedback via the focus groups. Additionally, exceptionally well-documented time and dose reporting allowed for a detailed exploration of SHHC-2.0 dose delivered and fidelity by each individual intervention component. Finally, the measurement of multiple implementation outcomes allowed for identification of both positive and negative aspects of the delivery process that highlight specific areas where additional implementation strategies may be needed to improve the delivery of SHHC-2.0.

## Conclusions

SHHC-2.0 had broad reach, was largely delivered as intended, and participants expressed high levels of satisfaction with SHHC-2.0 and its health benefits. Findings from this study can help provide guidance for researchers and practitioners who implement CVD or other health-related prevention programs, especially in rural areas. Future studies, including planned dissemination research for SHHC-2.0, should consider flexible and tailored implementation models as well as ensuring the efficiency and effectiveness of curricular components.

## Electronic supplementary material

Below is the link to the electronic supplementary material.

Supplementary Material 1

## Data Availability

The datasets used and/or analyzed during the current study are available from the corresponding author on reasonable request.

## Abbreviations

BMI: Body Mass Index
CVD: Cardiovascular disease
HEART: Healthy Eating and Activity in Rural Towns
cRCT: Community Randomized Control Trial
SHHC-2.0: Strong Hearts, Healthy Communities 2.0
