# Supplementary material for: Process evaluation findings from Strong Hearts, Healthy Communities 2.0: a cardiovascular disease prevention intervention for rural women
Source: Int J Behav Nutr Phys Act. 2024 Oct 22;21:122. doi: 10.1186/s12966-024-01670-y (PMC11515669; doi:10.1186/s12966-024-01670-y)
Supplement: Supplementary file 1 — Supplementary Material 1 [file 12966_2024_1670_MOESM1_ESM.pdf]

## Strong Hearts, Healthy Communities Class Activities Overview

| | | Aerobic Exercise | Strength Training | Nutrition Topic | HEART Club | Program Leader Teaches | Group Discussion | Activity | On the Menu |
|---|---|---|---|---|---|---|---|---|---|
| Icon indicates that activity or topic is part of the class. | | ✓ | ✓ | ✓ | ✓ | ✓ | ✓ | ✓ | ✓ |
| **Class 1** | **Welcome** | ✓ | | | | ✓ | | ✓ | ✓ |
| **Class 2** | **Where You Are and Where You Want To Be Part 1** | ✓ | ✓ | ✓ | | ✓ | | ✓ | |
| **Class 3** | **Physical Activity and Heart Disease in Women** | ✓ | | | | ✓ | ✓ | ✓ | ✓ |
| **Class 4** | **Healthy Eating: Fruits and Vegetables** | ✓ | ✓ | ✓ | | ✓ | ✓ | | ✓ |
| **Class 5** | **Weight Management and Fitness** | ✓ | ✓ | | | ✓ | | ✓ | |
| **Class 6** | **Healthy Eating: Fats and Proteins** | ✓ | ✓ | ✓ | | ✓ | ✓ | | ✓ |
| **Class 7** | **Strength Training Essentials** | ✓ | ✓ | | | ✓ | ✓ | ✓ | |
| **Class 8** | **Healthy Eating: Grains and Dairy** | ✓ | ✓ | ✓ | | ✓ | ✓ | | |
| **Class 9** | **Remodeling Your Home Environment Part 1: Making the Healthy Choice the Easy Choice** | ✓ | ✓ | ✓ | | | | ✓ | ✓ |
| **Class 10** | **Remodeling Your Home Environment Part 2: Making the Healthy Choice the Easy Choice** | ✓ | ✓ | ✓ | | ✓ | ✓ | ✓ | |
| **Class 11** | **Healthy Eating Away From Home** | ✓ | ✓ | ✓ | | | ✓ | ✓ | |
| **Class 12** | **Healthier Recipes** | ✓ | ✓ | ✓ | | ✓ | | ✓ | ✓ |

| | |
|---|---|
| Class 13 | Healthy Drinking and Emotional Eating |
| Class 14 | Putting it all Together: Meal Planning Part 1 |
| Class 15 | Putting it all Together: Meal Planning Part 2 |
| Class 16 | Physical Activity |
| Class 17 | Healthy Eating: Snacks and Treats |
| Class 18 | HEART Club: Introduction |
| Class 19 | HEART Club: Community Assessment Part 1 |
| Class 20 | HEART Club: Community Assessment Part 2 |
| Class 21 | Healthy Eating and Cooking Part 1 |
| Class 22 | Healthy Eating and Cooking Part 2 |
| Class 23 | Healthy Eating: Dinner |
| Class 24 | HEART Club: Issue Selection |
| Class 25 | HEART Club: Purpose |
| Class 26 | Where You Are and Where You Want To Be Part 2 |
| Class 27 | Learning About Where You Shop for Food |
| Class 28 | Heart-Healthy Grocery Shopping |
| Class 29 | HEART Club: Unified Message and Asset Mapping |
| Class 30 | Putting it all Together: Meal Planning Part 3 |
| Class 31 | HEART Club: Benchmarks |

| | |
|---|---|
| **Class 32** | **HEART Club: Action Planning** |
| **Class 33** | **Mindful Eating and Activity** |
| **Class 34** | **HEART Club: Identifying Stakeholders** |
| **Class 35** | **Community Health and Well-Being** |
| **Class 36** | **HEART Club: Check-In** |
| **Class 37** | **HEART Club: Future Directions Part 1** |
| **Class 38** | **More Mindful Eating** |
| **Class 39** | **Emotional Eating** |
| **Class 40** | **HEART Club: Future Directions Part 2** |
| **Class 41** | **Social Environment** |
| **Class 42** | **HEART Club: HEART Charter** |
| **Class 43** | **Supports and Barriers** |
| **Class 44** | **Sustaining Eating Well** |
| **Class 45** | **Sustaining Physical Activity** |
| **Class 46** | **Revisiting Stress Reduction** |
| **Class 47** | **Problem-Solving Process and Action Planning: 6 weeks, 6 months, and 1 year** |
| **Class 48** | **Celebration of Completion** |
